# Improving the Generalizability of Convolutional Neural Network-Based Segmentation on CMR Images

**DOI:** 10.3389/fcvm.2020.00105

**Published:** 2020-06-30

**Authors:** Chen Chen, Wenjia Bai, Rhodri H. Davies, Anish N. Bhuva, Charlotte H. Manisty, Joao B. Augusto, James C Moon, Nay Aung, Aaron M. Lee, Mihir M. Sanghvi, Kenneth Fung, Jose Miguel Paiva, Steffen E. Petersen, Elena Lukaschuk, Stefan K. Piechnik, Stefan Neubauer, Daniel Rueckert

**Affiliations:** ^1^Biomedical Image Analysis Group, Department of Computing, Imperial College London, London, United Kingdom; ^2^Data Science Institute, Imperial College London, London, United Kingdom; ^3^Department of Brain Sciences, Imperial College London, London, United Kingdom; ^4^Institute of Cardiovascular Science, University College London, London, United Kingdom; ^5^Department of Cardiovascular Imaging, Barts Heart Centre, St Bartholomew's Hospital, London, United Kingdom; ^6^NIHR Biomedical Research Centre at Barts, Queen Mary University of London, London, United Kingdom; ^7^NIHR BRC Oxford, Division of Cardiovascular Medicine, Radcliffe Department of Medicine, University of Oxford, London, United Kingdom

**Keywords:** artificial intelligence, deep learning, neural network, cardiac MR image segmentation, model generalization, cardiac image analysis

## Abstract

**Background:** Convolutional neural network (CNN) based segmentation methods provide an efficient and automated way for clinicians to assess the structure and function of the heart in cardiac MR images. While CNNs can generally perform the segmentation tasks with high accuracy when training and test images come from the same domain (e.g., same scanner or site), their performance often degrades dramatically on images from different scanners or clinical sites.

**Methods:** We propose a simple yet effective way for improving the network generalization ability by carefully designing data normalization and augmentation strategies to accommodate common scenarios in multi-site, multi-scanner clinical imaging data sets. We demonstrate that a neural network trained on a single-site single-scanner dataset from the UK Biobank can be successfully applied to segmenting cardiac MR images across different sites and different scanners without substantial loss of accuracy. Specifically, the method was trained on a large set of 3,975 subjects from the UK Biobank. It was then directly tested on 600 different subjects from the UK Biobank for intra-domain testing and two other sets for cross-domain testing: the ACDC dataset (100 subjects, 1 site, 2 scanners) and the BSCMR-AS dataset (599 subjects, 6 sites, 9 scanners).

**Results:** The proposed method produces promising segmentation results on the UK Biobank test set which are comparable to previously reported values in the literature, while also performing well on cross-domain test sets, achieving a mean Dice metric of 0.90 for the left ventricle, 0.81 for the myocardium, and 0.82 for the right ventricle on the ACDC dataset; and 0.89 for the left ventricle, 0.83 for the myocardium on the BSCMR-AS dataset.

**Conclusions:** The proposed method offers a potential solution to improve CNN-based model generalizability for the cross-scanner and cross-site cardiac MR image segmentation task.

## 1. Introduction

Automatic cardiac segmentation algorithms provide an efficient way for clinicians to assess the structure and function of the heart from cardiac magnetic resonance (CMR) images for the diagnosis and management of a wide range of abnormal heart conditions (1). Recently, convolutional neural network (CNN)-based methods have become state-of-the-art techniques for automated cardiac image segmentation (1, 2). However, related work (3) has shown that the segmentation accuracy of a CNN may degrade if the network is directly applied to images collected from different scanners or sites. For instance, CMR images from different scanners using different acquisition protocols can exhibit differences in terms of noise levels, image contrast, and resolution (4–6). Moreover, images coming from different sites may comprise different population demographics in terms of cardiovascular diseases, resulting in the clinically appreciable difference not only in cardiac morphology but also in image quality (e.g., irregular heartbeat can affect image quality) (7–9). Thus, a CNN learned from a limited dataset may not be able to generalize over subjects with heart conditions outside of the training set. All these differences pose challenges for deploying CNN-based image segmentation algorithms in real-world practice.

In general, a straightforward way to address this problem is to fine-tune a CNN learned from one dataset (source domain) with additional labeled data from another dataset (target domain). Nevertheless, collecting sufficient pixel-wise labeled medical data for every scenario can be difficult, since it requires domain-specific knowledge and intensive labor to perform manual annotation. To alleviate the labeling cost, unsupervised deep domain adaptation (UDDA) approaches have been proposed (10). Compared to fine-tuning, UDDA does not require labeled data from the target domain. Instead, it only uses either feature-level information (11–13) or image-level information (13) to optimize the network performance on the target domain. However, these methods usually require hand-crafted hyper-parameter tuning for each scenario, which may be difficult to scale to highly heterogeneous datasets. Therefore, it is of great interest to explore how to learn a network that can be successfully applied to other datasets without the requirement of additional model tuning.

In this paper, we investigate the possibility of building a generalizable model for cardiac MR image segmentation, given a training set from only one scanner in a single site. Instead of fine-tuning or adapting to get a new model for each particular scenario, our goal is to find a generalizable solution that can analyse “real-world” test images collected from multiple sites and scanners. These images consist of various pathology and cardiac morphology that may not be present in the training set, reflecting the complexity of a real-world clinical setting. To achieve this goal, we choose the U-Net (14) as the fundamental CNN architecture, which is the most popular network for medical image segmentation. We apply this network to segment the cardiac anatomy from CMR images (short-axis view), including the left ventricle (LV), the myocardium (MYO), and the right ventricle (RV). An image pre-processing pipeline is proposed to normalize images across sites before feeding them to the network in both training and testing stages. Data augmentation is employed in the pipeline during the training to improve the generalization ability of the network. Although there has been a number of works (15, 16) which have already applied data normalization and data augmentation in their pipelines, these methods are particularly designed for one specific dataset and the importance of applying data augmentation for model generalization ability across datasets is less explored. Here we demonstrate that the proposed data normalization and augmentation strategies can greatly improve the model performance in the cross-dataset setting (section 4.2). The main contributions of the work are as follows:

- To the best of our knowledge, this is the first work to explore the generalizability of CNN-based methods for cardiac MR image multi-structure segmentation, where the training data is collected from a **single scanner**, but the test data comes from **multiple scanners** and **multiple sites**.
- The proposed pipeline which employs data normalization and data augmentation (section 3.4) is simple yet efficient and can be applied to training and testing of many state-of-the-art CNN architectures to improve the model segmentation accuracy across domains without necessarily sacrificing the accuracy in the original domain. Experiment results show that the proposed segmentation method is capable of segmenting multi-scanner, multi-vendor, and multi-site datasets (sections 4.3, 4.4).
- Our work reveals that significant cardiac shape deformation caused by cardiac pathologies (section 4.5), low image quality (section 4.5), and inconsistent labeling protocols among different datasets (section 5) are still major challenges for generalizing deep learning-based cardiac image segmentation algorithms to images collected across different sites, which deserve further study.

## 2. Related Work

There have been a great number of works which develop sophisticated deep learning approaches to perform CMR image segmentation tasks on a specific dataset (1, 3, 15, 16). While these models can achieve overall high accuracy over the samples from the same dataset, only a few have been validated in cross-dataset settings. Table 1 shows a list of related works that demonstrate the segmentation performance of their proposed method by first training a model from one set (source domain) and then testing it on other datasets (target domain). However, these approaches requires re-training or fine-tuning to improve the performance on the target domain in a fully supervised fashion. To the best of our knowledge, there are few studies reported in the literature which investigate the generalization ability of the cardiac segmentation networks that can directly work across various sites.

**Table 1:** Related work that applies CNN-based CMR image segmentation models across multiple datasets.

**Methods**	**Target domain ≠ Source domain**	**Need Finetuning**	**Test on**	**Total size of test set (s)**
Tran (16)	Yes	Yes	LV/MYO/RV separately	<200
Bai et al. (3)	Yes	Yes	LV+MYO+RV	<100
Khened et al. (17)	Yes	No	MYO	<200
Our work	Yes	No	LV+MYO+RV	699

One work (18) in this line of research has been recently presented, which integrates training samples from multiple sites and multiple vendors (18) to improve segmentation performance across sites. Their results show that the best segmentation performance on their multi-scanner test set was achieved when the data used for training and testing are from the same scanners. Nevertheless, their solution requires collecting annotated data from multiple vendors and sites. For deployment, this may not always be practical because of the high data collection and labeling costs as well as data privacy issues.

Another direction to improve model generalization is to optimize the CNN architecture. In the work of Khened et al. (17), the authors proposed a novel network structure with residual connections to improve the network generalizability. They pointed out that networks with a large number of parameters may easily suffer from over-fitting problem with limited data (17). They demonstrated that their light-weight network trained on a limited dataset outperformed the U-Net (14), achieving higher accuracy on LV, myocardium, and RV. Moreover, model generalization was demonstrated by directly testing this network (without any re-training or fine-tuning) on the LV-2011 dataset (19). As a result, this model produced comparable results to the results from a network that had been trained on the LV-2011, achieving a high mean Dice score for the myocardium (0.84). However, because of the lack of RV labels in their test set, their network's generalization ability for the RV segmentation task is unclear. In fact, segmenting the RV is considered to be harder than segmenting the LV because the RV has a more complex shape with higher variability across individuals, and its walls are thinner, making it harder to delineate from its surroundings. Because of the high shapes variability and complexity, it is more difficult to generalize a model to segment the RV across domains.

In this study, we evaluate the generalizability of the proposed method not only on the cardiac left ventricle segmentation but also on the right ventricle segmentation. Different from the works in Tao et al. (18) and Khened et al. (17), the proposed method demonstrates model generalizability in a more challenging but realistic setting: our training data was collected from only one scanner (most of them are healthy subjects) while test data was collected from various unseen sites and scanners, which covers a wide range of pathologies, reflecting the spectrum of clinical practice.

## 3. Materials and Methods

### 3.1. Data

Three datasets are used in this study and the general descriptions of them are summarized in Table 2.

**Table 2:** General descriptions of the three datasets.

**Name**	**Number of subjects**	**Cohort**	**Sites**	**Scanners**	**Image spatial resolution**
UKBB	4,875	General population	1	1.5 T, Aera, Siemens (100%)	in-plane resolution: 1.8 mm^2^ /pixel; slice thickness: 8 mm
ACDC	100	Without cardiac disease (20%); Dilated cardiomyopathy (20%); Hypertrophic cardiomyopathy (20%); Myocardial infarction with altered left ventricular ejection (20%); Abnormal right ventricle (20%)	1	1.5 T, Area, Siemens (67%) 3 T, Trio Tim, Siemens (33%)	in-plane resolution: 1.34–1.68 mm^2^ /pixel; slice thickness: 5–10 mm
BSCMR-AS	599	Aortic stenosis	6	1.5 T, Ingenia, Philips (5.2%); 1.5 T, Intera, Philips (17.9%); 1.5 T, Sonata, Siemens (6.2%); 1.5 T, Aera, Siemens (0.5%); 1.5 T, Avanto, Siemens (56.6%); 3 T, Achieva, Philips (0.7%); 3 T, Skyra, Siemens (3.8%); 3 T, Verio, Siemens (5.0%); 3 T, TrioTim, Siemens (4.2%);	in-plane resolution: 0.78–2.3 mm^2^; slice thickness: 5–10 mm

#### 3.1.1. UK Biobank Dataset

The UK Biobank (UKBB) is a large-scale data set that is open to researchers worldwide who wish to conduct a prospective epidemiological study. The UKBB study covers a large population, which consists of over half a million voluntary participants aged between 40 and 69 from across the UK. Besides, the UKBB study performs comprehensive MR imaging for nearly 100,000 participants, including brain, cardiac and whole-body MR imaging. An overview of the cohort characteristics can be found on the UK Biobank's website^1^. All CMR images we used in this study are balanced steady-state free precession (bSSFP) sequences, which were collected from one 1.5 Tesla scanner (MAGNETOM Aera, syngo MR D13A, Siemens, Erlangen, Germany). Detailed information about the imaging protocol can be found in Petersen et al. (20). Pixel-wise segmentations of three essential structures (LV, MYO, and RV) for both end-diastolic (ED) frames and end-systolic (ES) frames are provided as ground truth (21). Subjects in this dataset were annotated by a group of eight observers and each subject was annotated only once by one observer. After that, visual quality control was performed on a subset of data to assure acceptable inter-observer agreement.

#### 3.1.2. ACDC Dataset

The Automated Cardiac Diagnosis Challenge (ACDC) dataset is part of the MICCAI 2017 benchmark dataset for CMR image segmentation^2^. This dataset is composed of 100 CMR images, acquired using bSSFP imaging in breath hold with a retrospective or prospective gating (1). The patients covered in this study have been divided into five groups: dilated cardiomyopathy (DCM), hypertrophic cardiomyopathy (HCM), myocardial infarction with altered left ventricular ejection fraction (MINF), abnormal right ventricle (ARV), and patients without cardiac disease (NOR). Each group has 20 patients. Detailed information about the classification rules and the characteristics of each group can be found in the benchmark study (1) as well as its website (see footnote 2). All images were collected from one hospital in France. The LV, MYO, and RV in this dataset have been manually segmented for both ED frames and ES frames. Images in this dataset were labeled by two cardiologists with more than 10 years of experience^3^.

#### 3.1.3. BSCMR-AS Dataset

The British Society of Cardiovascular Magnetic Resonance Aortic Stenosis (BSCMR-AS) dataset (22) consists of CMR images of 599 patients with severe aortic stenosis (AS), who had been listed for surgery. Images were collected from *six* hospitals across the UK with nine types of scanners (see Table 2). Specifically, these images are bSSFP sequences, which were acquired using standard imaging protocols (22). Although the primary pathology is AS, several other pathologies coexist in these patients (e.g., coronary artery disease, amyloid) and have led to a variety of cardiac phenotypes including left ventricular hypertrophy, left ventricular dilatation and regional infarction (22). A more detailed report on patients characteristics can be found in Musa et al. (22). In this dataset, no subjects were excluded due to arrhythmi. A significant amount of diversity in image appearance and image contrast can be observed in this dataset. Different from the above two data sets, images in this dataset are partially labeled. Only the left ventricle in ED frames and ES frames, as well as the myocardium in ED frames, have been annotated manually. The contours on each slice were refined by an expert.

#### 3.1.4. Ethics Approval and Consent to Participate

The UK Biobank data has approval from the North West Research Ethics Committee (REC reference: 11/NW/0382). The ACDC data is a publicly available dataset for cardiac MR image analysis which has approval from the local ethics committee of Hospital of Dijon (France)^4^. The BSCMR-AS data has approval from the UK National Research Ethics Service (REC reference:13/NW/0832), and has been conformed to the principles of the Declaration of Helsinki. All patients gave written informed consent.

### 3.2. Training Set and Test Sets

In this study, we use the UKBB dataset for training and intra-domain testing, and use the ACDC data and BSCMR-AS dataset for cross-domain testing. Following the same data splitting strategy in Bai et al. (3), we split the UKBB dataset into three subsets, containing 3,975, 300, and 600 subjects for each set. Specifically, 3,975 subjects were used to train the neural network while 300 validation subjects were used for tracking the training progress and avoid over-fitting. The subset consisting of remaining 600 subjects was used for evaluating models' performance in the intra-domain setting. In addition, we directly tested this trained network on the other two unseen cross-domain datasets: ACDC and BSCMR-AS datasets *without any further re-training or fine-tuning process*. The diversity of pathology observed in the ACDC dataset and the diversity of scanners and cardiac morphologies in the BSCMR-AS set make them ideal test sets for evaluating the proposed method's segmentation performance across sites.

### 3.3. Network Architecture

**Figure 1:**
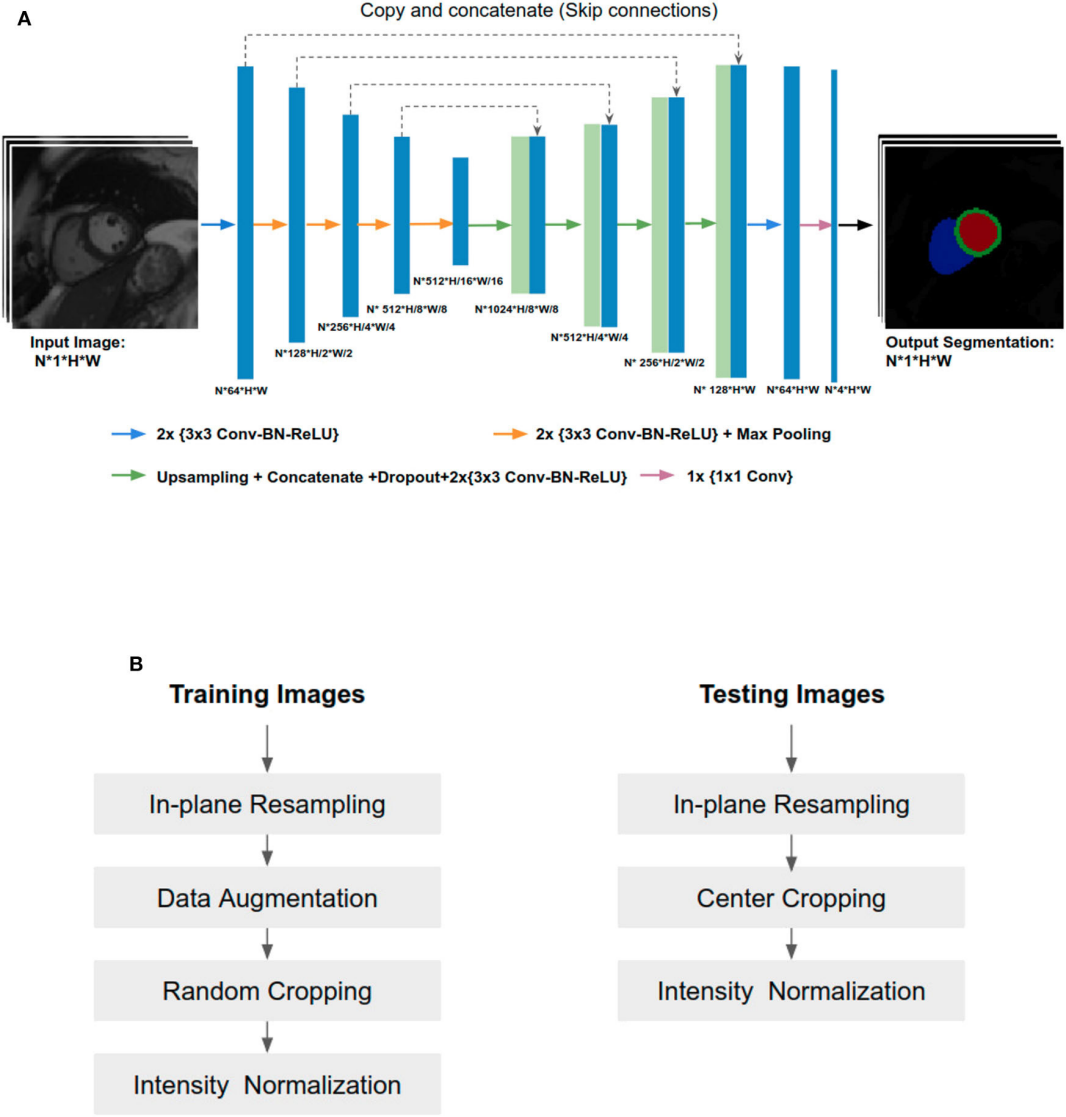
**(A)** Overview of the network structure. Conv, Convolutional layer; BN, Batch normalization; ReLU, Rectified linear unit. The U-Net takes a batch size of N 2D CMR images as input at each iteration, learning multi-scale features through a series of convolutional layers, max-pooling operations. These features are then combined through upsampling and convolutional layers from coarse to fine scales to generate pixel-wise predictions for the four classes (background, LV, MYO, RV) on each slice. **(B)** Image pre-processing during training and testing.

In this paper, the U-Net architecture (14) is adopted to perform the cardiac multi-structure segmentation task since it is the most successful and commonly used architecture for biomedical segmentation. The structure of our network is illustrated in Figure 1A. The network structure is as same as the one proposed in the original paper (14), except for two main differences: (1) we apply batch normalization (BN) (23) after each hidden convolutional layer to stabilize the training; (2) we apply dropout regularization (24) after each concatenating operation to avoid over-fitting and encourage generalization.

While both 2D U-Net and 3D U-Net architectures can be used to solve volumetric segmentation tasks (15, 25), we opt for 2D U-Net for several reasons. Firstly, performing segmentation tasks in a 2D fashion allows the network to work with images even if they have different slice thickness or have severe respiratory motion artifacts between the slices (which is not uncommon). Secondly, 3D networks require much more parameters than 2D networks. Therefore, it is more memory-consuming and time-consuming to train a 3D network than a 2D one. Thirdly, the manual annotation for images in the three datasets were done in 2D (slice-by-slice) rather than 3D. Thus, it is natural to employ a 2D network rather than a 3D network to learn segmentation from those 2D labels.

### 3.4. Training and Testing Pipeline

Since training images and testing images in this study were collected from various scanners, it is vital to normalize the input images before feeding them into the network. Figure 1B shows an overview of the pipeline for image pre-processing during training and testing. Specifically, we employ image resampling and intensity normalization to normalize images in both the training and testing stages while online data augmentation is applied for improving the model generalization ability during the training process.

#### 3.4.1. Image Resampling

Observing that the size of the heart in images with different resolution can vary significantly, we propose to perform image resampling both in the training and testing phases before cropping. The main advantage is that after image resampling, the proportion of the heart and the background is relatively consistent, which can help to reduce the task complexity of the follow-up segmentation. However, image re-sampling is not a lossless operation, and different interpolation kernels can also affect the quality of reconstructed images (26). In the experiments, we resampled all the images to a standard resolution of 1.25 × 1.25 mm^2^, which is a median value of the pixel spacings in our datasets. Following Isensee et al. (25), images are resampled using the bilinear interpolation and the label maps are resampled using nearest-neighbor interpolation.

Here we only perform image resampling within the short-axis plane, without changing the slice thickness along the z-axis. This is consistent with the preprocessing step in other existing 2D CNN-based approaches for cardiac image segmentation (1, 15, 25). Also, in our experiments, we found that the slice thickness does not have a significant impact on the model performance. The model performs consistently well across test images of different slice thicknesses (see Table S1), while it was only trained using images of 8 mm slice thickness.

#### 3.4.2. Data Augmentation

Data augmentation has been widely used when training convolutional neural networks for computer vision tasks on natural images. While different tasks may have different domain-specific augmentation strategies, the common idea is to enhance model's generalization by artificially increasing the variety of training images so that the training set distribution is more close to the test set population in the real world.

In this study, the training dataset is augmented in order to cover a wide range of geometrical variations in terms of the heart pose and size. To achieve this goal, we apply:

- Random horizontal and vertical flips with a probability of 0.5 to increase the variety of image orientation;
- Random rotation to increase the diversity of the heart pose. The range of rotation is determined by a hyper-parameter search process. As a result, each time, the angle for augmentation is randomly selected from [−30, +30];
- Random image scaling with a scale factor *s*: *s*∈[0.7, 1.4] to increase variations of the heart size;
- Random image cropping. The random cropping crops images to acceptable sizes required by the network structure while implicitly performing random shifting to augment data context variety without black borders. Note that cropping is done after all other image augmentations. As a consequence, all images are cropped to the same size of 256 × 256 before being sent to the network.

We also experimented with contrast augmentation (27) (random gamma correction where the gamma value is randomly chosen from a certain range) to increase image contrast variety, but only minor improvements were found in the experiments. Therefore, it is not included in the pipeline. For each cropped image, intensity normalization with a mean of 0 and a standard deviation of 1 is performed, which is a common practice for training deep neural networks.

#### 3.4.3. Training

After pre-processing, batches of images are fed to the network for training. To track the training progress, we also use a subset (validation set) from the same dataset to validate the performance of the segmentation and to identify possible over-fitting. Specifically, we apply the same data augmentation strategy on both the training and validation sets and record the average accuracy (mean intersection of union between predicted results and ground truth) on the validation set for each epoch. The model with the highest accuracy is selected as the best model. This selection criterion works as early stopping and has the benefit of allowing the network to explore if there is further opportunity to generalize better before it reaches to the final epoch.

#### 3.4.4. Testing

For testing, 2D images extracted from volume data are first re-sampled and centrally cropped to the same size as the one of the training images. Again, intensity normalization is performed on each image slice which is then passed into the network for inference. After that, bilinear up-sampling or down-sampling is performed on the outputs of the network to recover the resolution back to the original one. Finally, each pixel of the original image is assigned to the class that has the highest probability among the four classes (background, LV, myocardium, RV). As a result, a final segmentation map for one input image is generated.

### 3.5. Implementation Details

During training, a random batch of 20 2D short-axis slices were fed into the network for each iteration after data pre-processing. The dropout rate for each dropout layer is set to be 0.2. In every iteration, cross entropy loss was calculated to optimize the network parameters through back-propagation. Specifically, the stochastic gradient descent (SGD) method was used during the optimization, with an initial learning rate of 0.001. The learning rate was decreased by a factor of 0.5 every 50 epochs. The method was implemented using Python and PyTorch. We trained the U-Net for 1,000 epochs in total which took about 60 hours on one NVIDIA Tesla P40 GPU using our proposed training strategy. During testing, the computation time for segmenting one subject is less than a second.

### 3.6. Evaluation Metrics

The performance of the proposed method was evaluated using the Dice score (3D version) which was also used in the ACDC benchmark study (1, 3). The Dice score evaluates the overlap between automated segmentation *A* and manual segmentation *B*, which is defined as: $\text{Dice}=\frac{2|A\cap B|}{\left|A|+|B\right|}.$ The value of a Dice score ranges from 0 (no overlap between the predicted segmentation and its ground truth) to 1 (perfect match).

We also compared the volumetric measures derived from our automatic segmentation results and those from manual ones (see section 4.6), since they are essential for cardiac function assessment. Specifically, for each manual ground truth mask and its corresponding automatic segmentation mask, we calculated the volumes of LV and RV at ED frames and ES frames, as well as the mass of myocardium estimated at ED frames. The myocardium mass around the LV is estimated by multiplying the LV myocardial volume with a density of 1.05 g/mL. After that, Bland-Altman analysis and correlation analysis for each pair were conducted. Of note, for Bland-Altman analysis, we removed the outlying mean values that fall outside the range of 1.5 × IQR (interquartile range) in order to avoid the standard deviation of mean difference being biased by extremely large values. These outliers are often associated with poor image quality. As a result, <3% subjects were removed in each comparison.

The statistical analysis was performed using python with public packages: *pandas*^5^, *scipy.stats*^6^, and *statsmodel*^7^.

## 4. Results

To demonstrate the improvement of model generalization performance, we directly tested the proposed segmentation method across three sets: the UKBB test set, the ACDC set, and the BSCMR-AS set, and compared the segmentation accuracy to the performance of the segmentation method in our previous work (3). Specifically, in Bai et al. (3), a fully convolutional neural network (FCN) was proposed, which was specifically designed to automatically segment a large scale of scans for the same cohort study (i.e., UKBB study) with maximum accuracy whereas the proposed method in our study focuses on improving the robustness of the neural network-based segmentation method (using the same UKBB training set as training data) for data from different domains (e.g., non-UKBB data). The comparison results are shown in Table 3.

**Table 3:** Comparison results of segmentation performance between a baseline method and the proposed method across three test sets.

		**UKBB test set (*****n*** **= 600)**			**ACDC set (*****n*** **= 100)**			**BSCMR-AS set (*****n*** **= 599)**	
**Method**	**Training set**	**LV**	**MYO**	**RV**	**LV**	**MYO**	**RV**	**LV**	MYO*
Bai et al. (3)	UKBB (*n* = 3,975)	0.94 (0.04)	0.88 (0.03)	0.90 (0.05)	0.81 (0.22)	0.70 (0.20)	0.68 (0.31)	0.82 (0.21)	0.74 (0.17)
Ours	UKBB (*n* = 3,975)	0.94 (0.04)	0.88 (0.03)	0.90 (0.05)	0.90 (0.10)	0.81 (0.07)	0.82 (0.13)	0.89 (0.09)	0.83 (0.07)

*Both methods were trained using the same UKBB training set. The results were evaluated on three sets. Numbers listed in the table are the means and standard deviation of Dice scores*.

*The myocardium segmentation performance on the BSCMR-AS set was only evaluated on ED frames because of the lack of annotation at ES frames, whereas the performance on the other two datasets was evaluated on both ED and ES frames. For simplicity, Dice scores for the myocardium on the BSCMR-AS in the following tables were calculated in the same way without further illustration.

**Figure 2:**
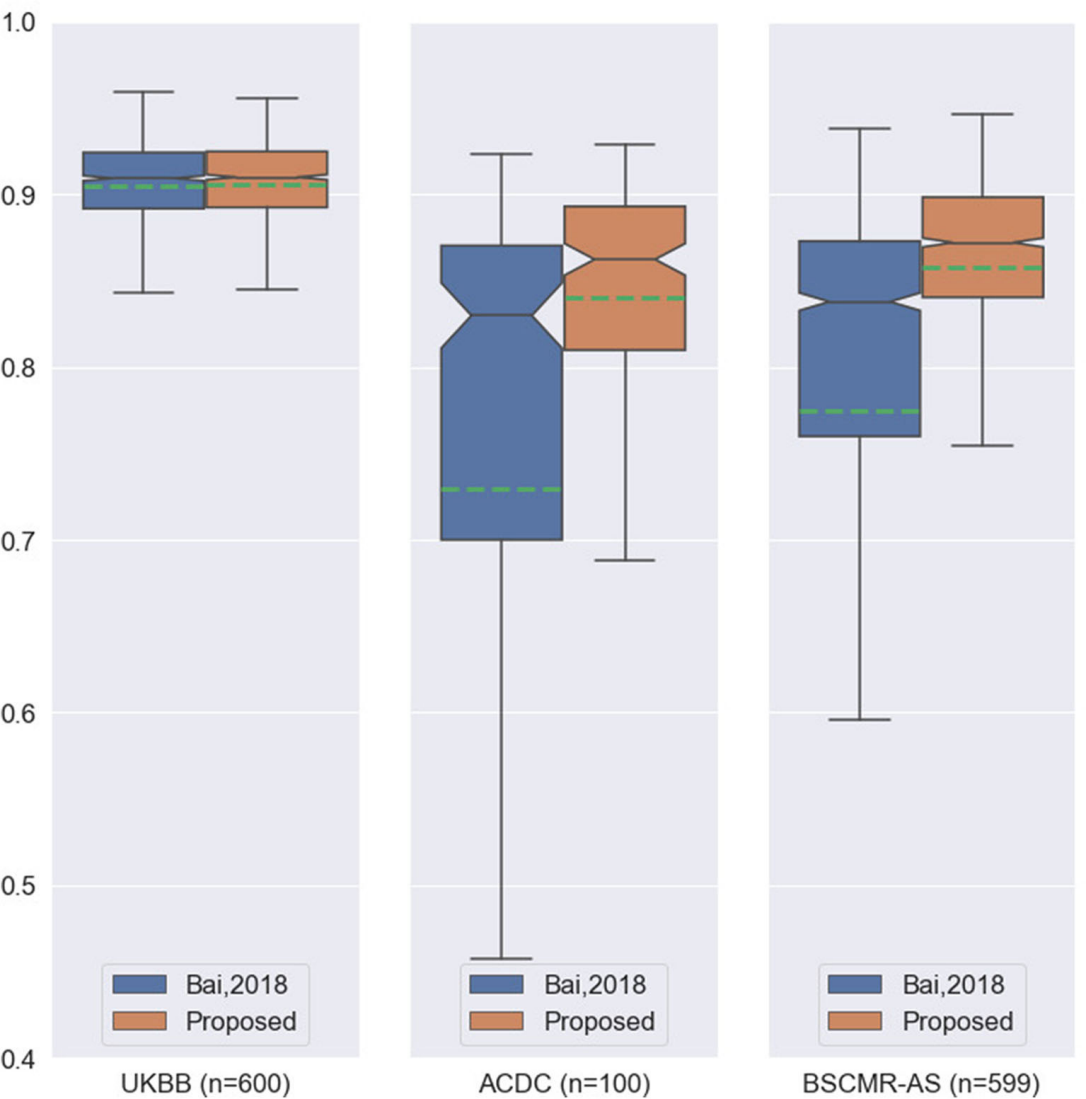
Boxplots of the average Dice scores between the results of our previous work (3) and the results of the proposed method on the three datasets. For simplicity, we calculate the average Dice score over the three structures (LV, MYO, RV) for each image in the three datasets. The boxplots in orange are the results of the proposed method whereas the boxplots in blue are the results of the previous work. The green dashed line in each boxplot shows the mean value of the Dice scores for the segmentation results on one dataset.

While both methods achieve very similar Dice scores on the intra-domain UKBB test set with high accuracy, the proposed method significantly outperforms the previous approach on the two cross-domain datasets: ACDC set and BSCMR-AS set. Compared to the results predicted using the method in Bai et al. (3) on the ACDC data, the proposed one achieves higher mean Dice scores for all of the three structures: LV (0.90 vs. 0.81), myocardium (0.81 vs. 0.70), and RV (0.82 vs. 0.68). On the BSCMR-AS dataset, the proposed method also yields higher average Dice scores for the LV cavity (0.89 vs. 0.82) and the myocardium (0.83 vs. 0.74). Figure 2 compares the distributions of Dice scores for the results obtained by the proposed method and the previous work. From the results, the boxplots of the proposed method are shorter than those of the previous method and have higher mean values, which suggests that the proposed method achieves comparatively higher overall segmentation accuracy with lower variance on the three datasets.

In order to identify what contributes to the improved performance, we further compare the proposed method with Bai et al. (3) in terms of methodology. Two main differences are spotted:

- **Network structure and capacity**. Compared to the U-Net we used in this study, FCN in Bai et al. (3) has a smaller number of filters at each level. For example, the number of convolutional kernels (filters) in the first layer of FCN is 16 whereas the one in the U-Net is 64. In addition, in the decoder part, FCN directly upsamples the featuremap from each scale to the finest resolution and concatenates all of them, whereas the U-Net adopts a hierarchical structure for feature aggregation.
- **Training strategy in terms of data normalization and data augmentation**. Compared to the image pre-processing pipeline in the previous work, the proposed pipeline adopts image resampling and random image flip augmentation in addition to the general data augmentation based on affine transformations.

In order to study the influence of the network structure as well as the data normalization and augmentation settings on model generalizability, extensive experiments were carried out and the results are shown in the next two sections.

### 4.1. The Influence of Network Structure and Capacity

To investigate the influence of network structure on model generalization, we trained three additional networks:

- FCN-16: the FCN network presented in Bai et al. (3) which has 16 filters in the first convolutional layer.
- FCN-64: a wider version of FCN where the number of filters in each convolutional layer is increased by 4 times.
- UNet-16: a smaller version of U-Net where the number of filters in each convolutional layer is reduced by four times. Same as FCN-16, it has 16 filters in the first layer.

All of them were trained using the same UKBB training set and with the same training hyperparameters. These networks were then compared to the proposed network (UNet-64).

Table 4 compares the performances of the four different networks over the three different test sets. It can be seen that while there is no significant performance difference among the four networks on the UKBB test set, small networks: UNet-16 and FCN-16 perform much more poorly than their wider versions: UNet-64 and FCN-64, on the ACDC set (see red numbers in Table 4). This may indicate that in order to accommodate more variety of data augmentation for generalization, the network requires a larger capacity. It is also worth noticing that UNet-64 outperforms FCN-64 on all of the three test sets, while UNet-64 contains fewer parameters than FCN-64. This improvement may result from U-Net's special architecture: skip connections with its step-by-step feature upsampling and aggregation. The results indicate that the network structure and capacity can affect the segmentation model generalizability across datasets.

**Table 4:** Cross-dataset segmentation performances of four different network architectures.

		**UKBB test set (*****n*** **= 600)**			**ACDC set (*****n*** **= 100)**			**BSCMR-AS set (*****n*** **= 599)**	
**Network structure**	**Num of conv weights (aprox.)**	**LV**	**MYO**	**RV**	**LV**	**MYO**	**RV**	**LV**	**MYO**
FCN-16	0.98 million	0.92 (0.04)	0.84 (0.04)	0.88 (0.05)	0.80 (0.20)	0.67 (0.19)	0.68 (0.27)	0.84 (0.14)	0.77 (0.11)
FCN-64	15.6 million	0.94 (0.04)	0.87 (0.03)	0.89 (0.05)	0.87 (0.12)	0.78 (0.11)	0.77 (0.17)	0.85 (0.12)	0.79 (0.10)
UNet-16	0.84 million	0.92 (0.04)	0.83 (0.04)	0.87 (0.05)	0.87 (0.12)	0.66 (0.14)	0.67 (0.22)	0.85 (0.11)	0.73 (0.11)
Ours (UNet-64)	13.4 million	**0.94 (0.04)**	**0.88 (0.03)**	**0.90 (0.05)**	**0.90 (0.10)**	**0.81 (0.07)**	**0.82 (0.13)**	**0.88 (0.09)**	**0.83 (0.07)**

*All the networks have been trained using the same UKBB training set with the proposed data normalization and augmentation strategy for 1,000 epochs. Results listed in the table are the means and standard deviation of the Dice scores evaluated on the three sets. Numbers in red denote mean Dice scores below 0.70, whereas numbers in the bold font style denote the highest mean Dice scores among the results of the four networks*.

### 4.2. The Influence of Different Data Normalization and Data Augmentation Techniques

In this section, we investigate the influence of different data normalization and augmentation techniques on the generalizability of the network, including image resampling (data normalization), scale, flip, and rotation augmentation (data augmentation). We focus on these four operations because convolutional neural networks are designed to be translation-equivariant (28) but they are not rotation-equivariant, nor scale and flip-equivariant (29, 30). This means that if we rotate the input, the networks cannot be guaranteed to produce the same predictions with the corresponding rotation, indicating that they are not robust to geometrical transformations on images. Current methods to improve these networks' ability to deal with rotation/flip/scale variations still heavily rely on data augmentation while intensity-level difference might be addressed by further doing domain adaptation techniques such as style transfer or adaptive batch normalization (31).

To investigate the influence of these four operations on model generalization, we trained additional three U-Nets using the UKBB training set, each of them was trained with the same settings except that only one operation was removed. To save the computational time for this ablation study, each network was trained for 200 epochs, which still took 10 h for each network since the training set from the UKBB dataset was considerably large (3,975 subjects). The test results on the UKBB test set, the ACDC dataset, and the BSCMR-AS dataset are shown in Table 5. It can be observed that while the results on the test data from the same domain (UKBB) with different settings do not vary much, there are significant differences on the other two test sets, demonstrating the importance of the four data augmentation operations. For example, image resampling increases the averaged Dice score from 0.673 to 0.783 for the RV segmentation on the BSCMR-AS set, whereas augmentation by scaling improves the mean Dice score from 0.596 to 0.750 for the RV on the ACDC set. The best segmentation performance over the three sets is achieved by combining all the four operations.

**Table 5:** Cross-dataset segmentation performances of U-Nets with different training configurations.

**Configurations**				**UKBB test set (*****n*** **= 600)**			**ACDC set (*****n*** **= 100)**			**BSCMR-AS set (*****n*** **= 599)**	
**Image resample**	**Rotation Aug**	**Flip Aug**	**Scale Aug**	**LV**	**MYO**	**RV**	**LV**	**MYO**	**RV**	**LV**	**MYO**
✓	✓	✓	✓	0.923 (0.041)	0.847 (0.038)	0.878 (0.048)	0.873 (0.101)	0.744 (0.104)	0.750 (0.187)	0.851 (0.113)	0.783 (0.095)
✗	✓	✓	✓	0.916 (0.046)	0.836 (0.041)	0.864 (0.053)	0.811 (0.179)	0.614 (0.186)	0.575 (0.270)	0.798 (0.172)	0.673 (0.162)
✓	✗	✓	✓	0.922 (0.042)	0.848 (0.038)	0.878 (0.050)	0.869 (0.117)	0.733 (0.117)	0.722 (0.210)	0.853 (0.118)	0.784 (0.093)
✓	✓	✗	✓	0.924 (0.041)	0.849 (0.037)	0.881 (0.049)	0.858 (0.115)	0.705 (0.142)	0.681 (0.266)	0.862 (0.110)	0.779 (0.092)
✓	✓	✓	✗	0.921 (0.047)	0.845 (0.039)	0.876 (0.050)	0.785 (0.188)	0.640 (0.187)	0.596 (0.279)	0.834 (0.148)	0.752 (0.125)

*All experiments were performed with the standard U-Net architecture: UNet-64. Each U-Net was trained using the same UKBB training set for 200 epochs to save computation. Statistics listed in the table are the means and standard deviation of the Dice scores evaluated on the three sets. Numbers in red are those mean Dice scores below 0.70*.

These results suggest that increasing variations regarding pixel spacing (image scale augmentation), image orientation (flip augmentation), heart pose (rotation augmentation) as well as data normalization (image resampling) can be beneficial to improve model generalizabilty over unseen cardiac datasets. While one may argue that there is no need to do image resampling if scale augmentation is performed properly during training, we found that image resampling can significantly reduce the complexity of real-world data introduced by heterogeneous image pixel spacings, such that training and testing data are more similar to each other, bringing benefits to both model learning and prediction. In the following sections, for the sake of simplicity, we will use “UKBB model” to refer to our best model (the U-Net which was trained using the UKBB training set with our proposed training strategy).

### 4.3. Segmentation Performance on Images From Different Types of Scanners

In this section, UKBB model's segmentation performance is analyzed according to different manufacturers (Philips and Siemens) and different magnetic field strengths (1.5 Telsa and 3 Telsa). The results on the two datasets (BSCMR-AS and ACDC) are listed in Table 6. For ACDC data, only the results regarding scans imaged using different magnetic strengths are reported since these scans are all from Siemens. Furthermore, results in the ACDC dataset with Dice scores below 0.50 are not taken into account for this evaluation. This is because the number of subjects from a 3T scanner in the ACDC is so small (33 subjects) that the averaged performance can be easily affected given only a few cases with extreme low Dice scores. Here, six subjects were excluded. The final results show that the model trained only using 1.5T Siemens data (UKBB data) could still produce similar segmentation performance on other Siemens and Philips data (top two rows in Table 6). Similar results are found on those images acquired from 1.5T scanners and those acquired from 3T scanners (see the bottom four rows in Table 6). This indicates that the proposed method has the potential to train a model capable of segmenting images across **various scanners** even if the training images are only from **one** scanner.

**Table 6:** Segmentation performance of the UKBB model across different scanners.

**Dataset**	**MRI scanner attributes**	**Scanners**	**No. of subjects**	**LV**	**MYO**	**RV**
BSCMR-AS	Manufactures	Philips	142	0.89 (0.07)	0.85 (0.04)	–
		Siemens	457	0.88 (0.10)	0.83 (0.08)	–
	Magnetic field strengths	1.5T	517	0.88 (0.09)	0.83 (0.09)	–
		3 T	82	0.88 (0.09)	0.84 (0.09)	–
ACDC	Magnetic field strengths	1.5T	65	0.89 (0.09)	0.81 (0.06)	0.80 (0.09)
		3 T	29	0.91 (0.06)	0.82 (0.05)	0.80 (0.08)

*Tests were performed on the BSCMR-AS dataset and ACDC dataset. This table presents the mean and standard deviation (numbers in the brackets) of the Dice score*.

### 4.4. Segmentation Performance on Images From Different Sites

We also evaluate the performance of the UKBB model across seven sites: one from ACDC data, six sites from BSCMR-AS data. Results are shown in Table 7. From the results, no significant difference is found when evaluating the LV and the myocardium segmentation performances among the seven sites (A-G) while the generalization performance for RV segmentation still needs further investigation when more data with annotated RV becomes available for evaluation.

**Table 7:** Segmentation performance of the UKBB model across different sites.

**Dataset**	**Site**	**No. of subjects**	**LV**	**MYO**	**RV**
ACDC	site A	100	0.91 (0.07)	0.81 (0.08)	0.82 (0.11)
BSCMR-AS	site B	28	0.88 (0.09)	0.83 (0.04)	–
	Site C	74	0.88 (0.09)	0.83 (0.04)	–
	Site D	150	0.89 (0.07)	0.85 (0.04)	–
	Site E	122	0.86 (0.11)	0.81 (0.08)	–
	Site F	64	0.88 (0.09)	0.84 (0.08)	–
	Site G	160	0.89 (0.09)	0.85 (0.08)	–

*This table presents the mean and the standard deviation (numbers in the brackets) of Dice scores for each site*.

### 4.5. Segmentation Performance on Images Belonging to Different Pathologies

**Figure 3:**
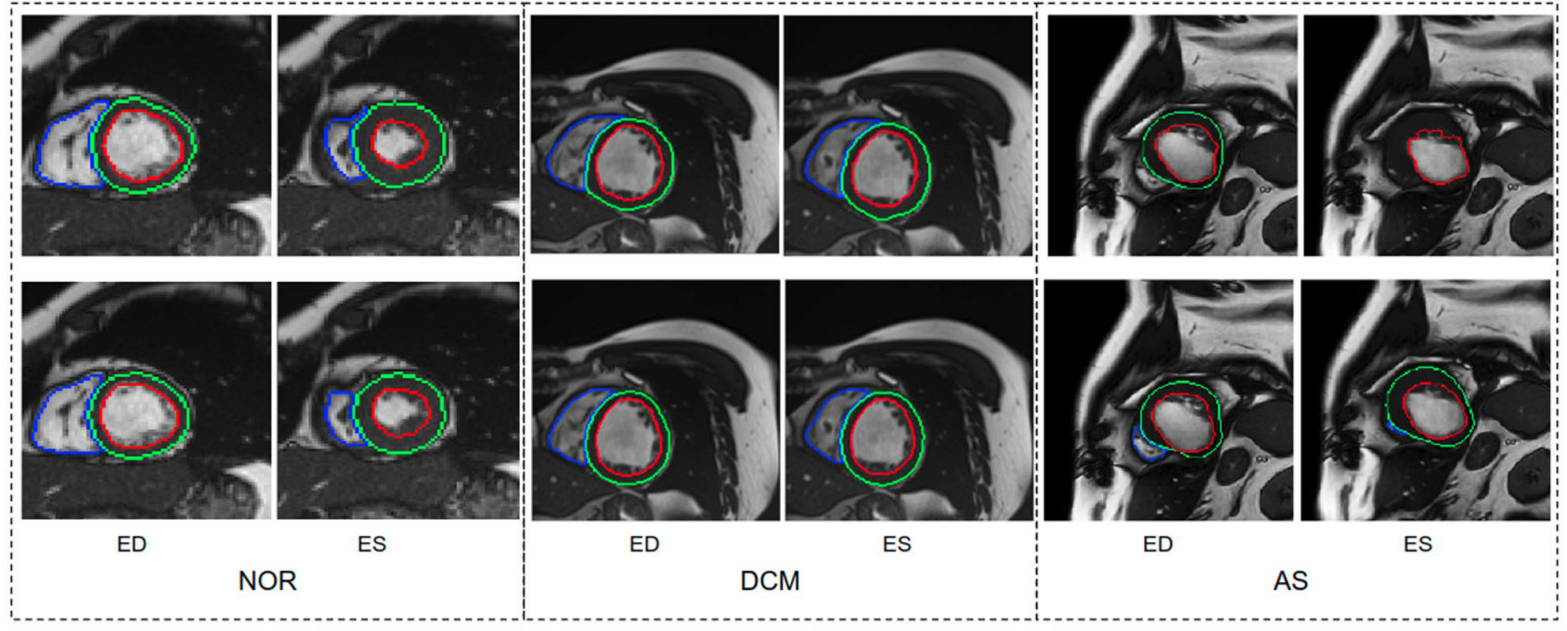
Visualization of good segmentation examples selected from three patient groups. NOR (without cardiac disease), DCM (dilated cardiomyopathy), AS (aortic stenosis). Row 1: Ground truth (manual annotations); row 2: predicted results by the UKBB model. Each block contains a slice from ED frame and its corresponding ES one for the same subject. This figure shows that the UKBB model produced satisfying segmentation results not only on healthy subjects but also on those DCM and AS cases with abnormal cardiac morphology. The AS example in this figure is a patient with aortic stenosis who previously had a myocardial infarction. Note that this AS case is from BSCMR-AS dataset where the MYO and RV on ES frames were not annotated by experts.

We further report the segmentation performance of the proposed method on five groups of pathological data and the group of normal subjects (NOR) (see Table 8). Surprisingly, the UKBB model achieves satisfying segmentation accuracy over the healthy group as well as DCM images and those images diagnosed with AS, indicating the model is capable of segmenting not only those with normal cardiac structures but also some abnormal cases with the cardiac morphological variations in those HCM images and AS images (see Figure 3).

**Table 8:** Segmentation performance of the UKBB model across the five groups of pathological cases and normal cases (NOR).

**Dataset**	**Group**	**No. of subjects**	**LV**	**MYO**	**RV**
ACDC	NOR	20	0.91 (0.05)	0.83 (0.04)	0.85 (0.14)
	DCM	20	0.94 (0.04)	0.81 (0.05)	0.82 (0.11)
	HCM	20	0.84 (0.12)	0.84 (0.03)	0.84 (0.08)
	MINF	20	0.92 (0.05)	0.81 (0.04)	0.78 (0.13)
	ARV	20	0.86 (0.13)	0.74 (0.11)	0.79 (0.16)
BSCMR-AS	AS	599	0.88 (0.09)	0.83 (0.07)	–

*This table presents the mean and standard deviation of the Dice score. Red numbers are those mean Dice scores below 0.80*.

**Figure 4:**
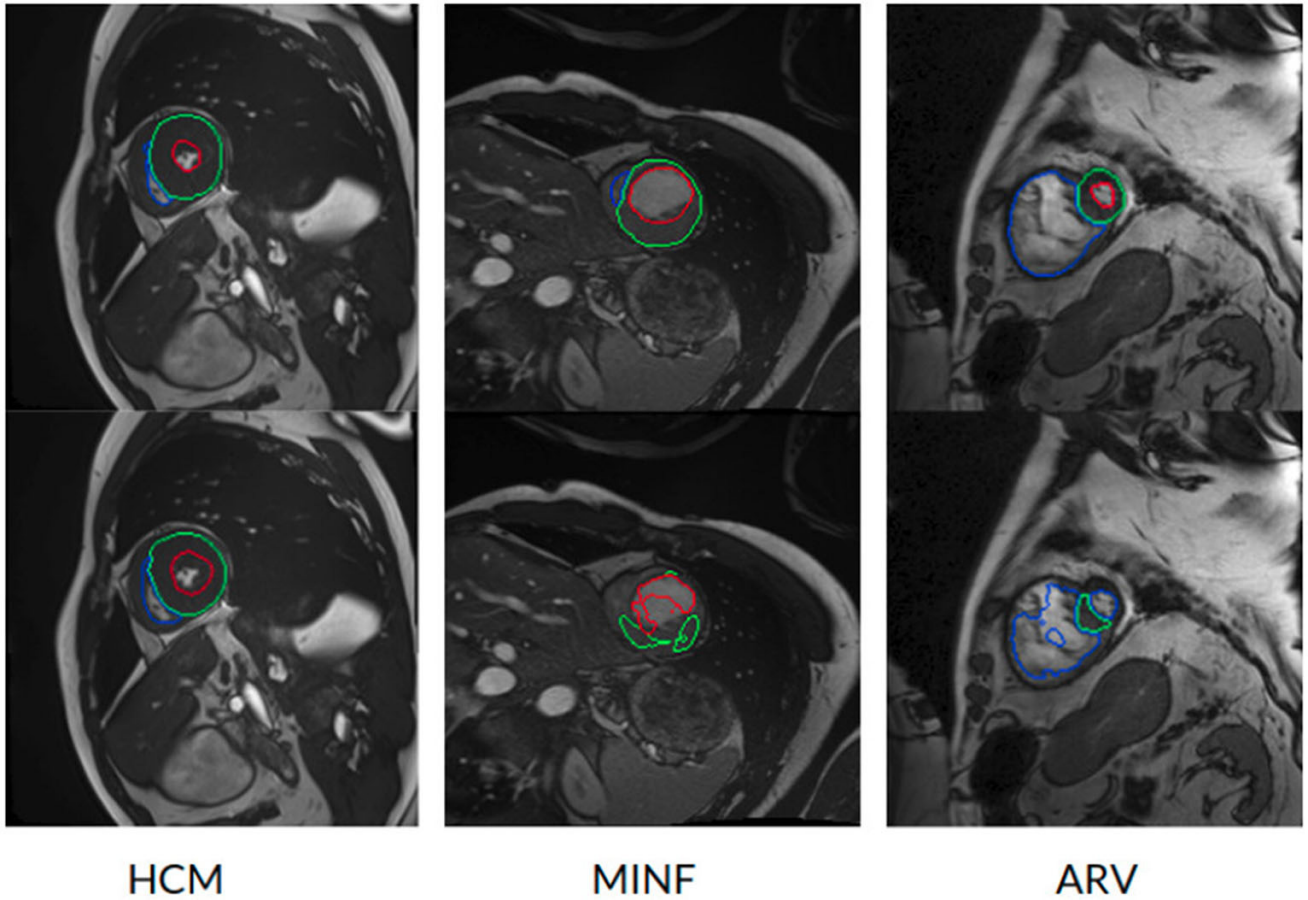
Examples of the worst cases that have pathological deformations. Row 1: Ground truth; row 2: predicted results by the UKBB model. HCM, hypertrophic cardiomyopathy; MINF, myocardial infarction with altered left ventricular ejection fraction; ARV, abnormal right ventricle. Column 1 shows that the UKBB model underestimates the myocardium in patients with HCM. Column 2 shows that the model struggles to predict the cardiac structure when certain sections of the myocardium are extremely thin. Column 3 shows a failure case where an extremely large right ventricle is shown in the image. All these images are from ACDC dataset.

However, the model fails to segment some of the other pathological images, especially those in the HCM, MINF, and ARV pathology groups where lower Dice scores are observed. For example, the mean Dice score for LV segmentation on HCM images is the lowest (0.84). Figure 4 demonstrates some of the worst cases produced by the proposed method. The first column in Figure 4, shows a failure case where the UKBB model underestimated the myocardium and overestimated the LV when a thickened myocardial wall is present in a patient with HCM. Also, the model struggles to segment cardiac structure on a patient with MINF which contains the abnormal myocardial wall with non-uniform thickness (the second column in Figure 4). Compared to images in the other four groups with pathology, images from patients with ARV seem to be more difficult for the model to segment as the model not only achieves a low mean Dice score on the RV (0.79) but also a low averaged value on the myocardium (0.74).

One possible reason for these unsatisfactory segmentation results might be the lack of pathological data in the current training set. In fact, the UKBB data only consists of a small amount of subjects with self-reported cardiovascular diseases, and the majority of the data are healthy subjects in middle and later life (3, 21, 32). This indicates that the network may not be able to “learn” the range of those pathologies that are seen in everyday clinical practice, especially those abnormalities which are not currently represented in the UKBB dataset.

#### 4.5.1. Failure Mode Analysis

**Figure 5:**
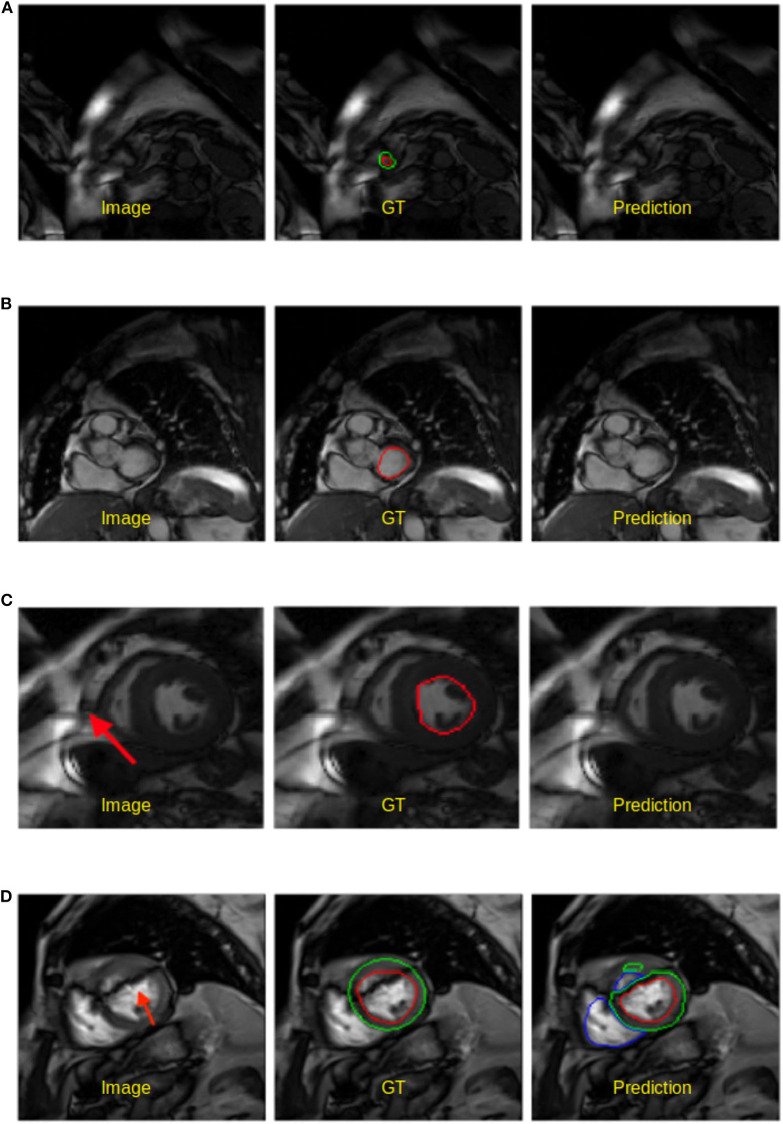
Examples of worst segmentation results found on challenging slices. Left: Image, middle: ground truth (GT), right: prediction from the UKBB model. **(A)** Failure to predict LV when the apical slice has a very small LV. **(B)** LV segmentation missing on the basal slice (ES frame). This sample is from the BSCMR-AS dataset where only the LV endocardial annotation is available. **(C)** Failure to recognize the LV due to a stripe of high-intensity noise around the cardiac chambers in this 1.5T image. This sample is an ES frame image from the BSCMR-AS dataset. **(D)** Failure to estimate the LV structure when unexpected strong dark artifacts disrupt the shape of the LV in this 3T image. Note that this image is an ED frame image from the BSCMR-AS dataset where RV was not annotated by experts.

We also visually inspected the images where the UKBB model produces poor segmentation masks. In general, there are two main failure modes we identified, apart from the failure found on the abnormal pathological cases which we have discussed above:

- **Apical and basal slices**. These slices are more error-prone than mid-ventricle slices, which has also been reported in Bernard et al. (1). Segmenting these slices is difficult because apical slices have extremely tiny objects which can be hard to locate and segment (see Figure 5A) whereas basal slices with complex structures increase the difficulty of identifying the contour of the LV (see Figure 5B).
- **Low image quality**. Images with poor quality are found both in 1.5T and 3T images (see Figures 5C,D). As reported in Rajiah and Bolen (4) and Alfudhili et al. (5), 1.5T images are more likely to have low image contrast than 3T images due to the low signal-to-noise (SNR) limits, whereas 3T images can have more severe imaging artifact issues than 1.5T images. These artifacts and noise can greatly affect the segmentation performance.

### 4.6. Statistical Analysis on Clinical Parameters

We further compare the proposed automatic method with manual approach on five clinical parameters, including the end-diastolic volume of LV (*LV*_*EDV*_), the end-systolic volume of LV (*LV*_*ESV*_), the left ventricular mass (*LVM*), the end-diastolic volume of right ventricle (*RV*_*EDV*_), and the end-systolic volume of RV (*RV*_*ESV*_).

**Figure 6:**
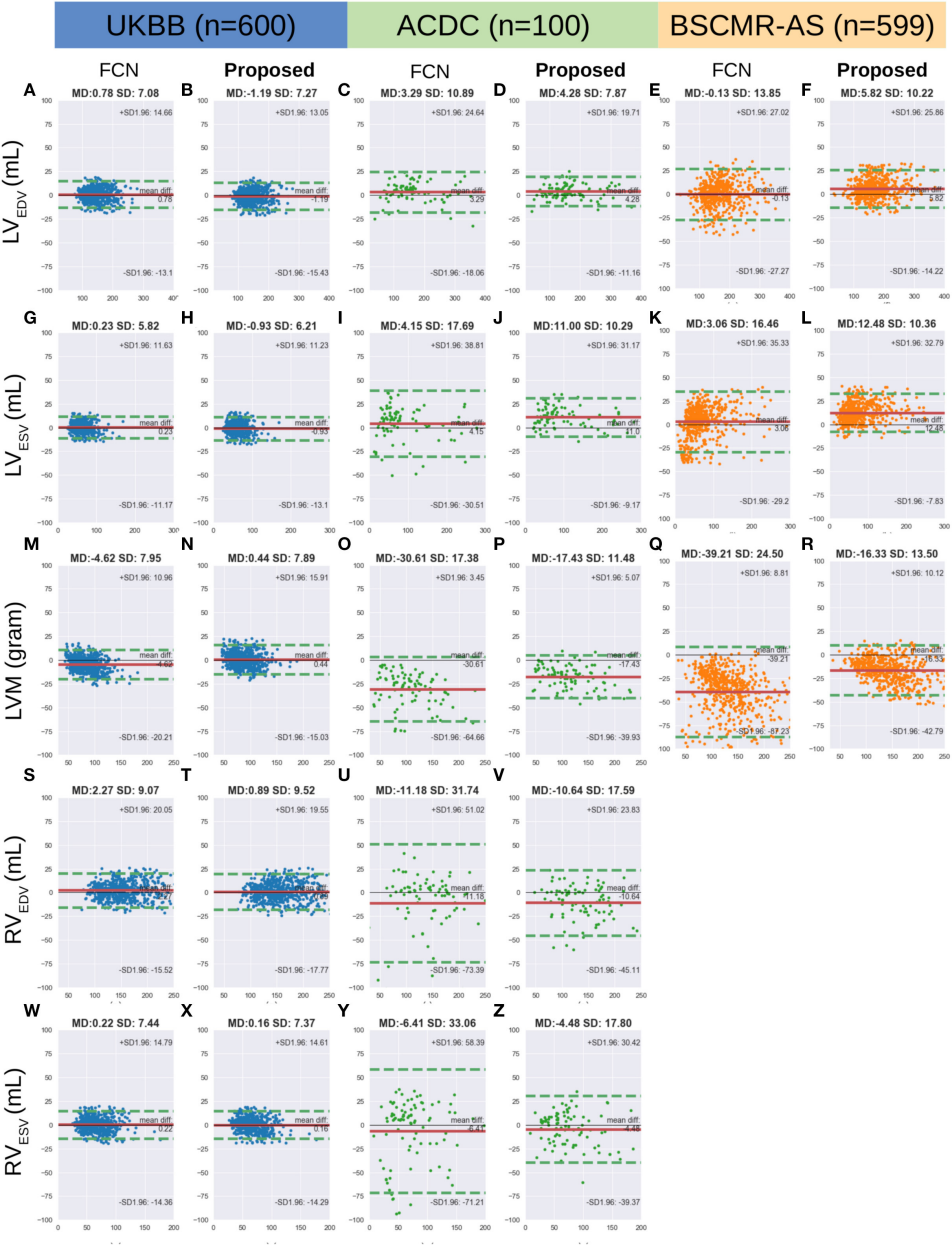
**(A-Z)** are Bland Altman plots (automatic-manual) for the three datasets. Agreement of clinical measurement from automatic and manual segmentations. Bland Altman plots (automatic-manual) are shown regarding the three sets. In each Bland-Altman plot, the x-axis denotes the average of two measurements whereas the y-axis denotes the difference between them. The solid line in red denotes the mean difference (bias) and the two dashed lines in green denote ±1.96 standard deviations from the mean. The title of each plot shows the mean difference (MD) and its standard deviation (SD) for each pair of measurements. FCN, the automatic method in our previous work (3); LV/RV, left/right ventricle; EDV/ESV, end-diastolic/systolic volume; LVM, left ventricular mass.

Figure 6 shows the Bland-Altman plots for the five clinical parameters on the three datasets. The Bland-Altman plot is commonly used for analysing agreement and bias between two measurements. Here, each column shows the comparison results between automated measurements and manual measurements for one particular parameter, including the mean differences (MD) with corresponding standard deviation (SD) and the limits of agreement (LOA). In addition, we also conducted the Bland-Altman analysis for the automatic method (FCN) in our previous work (3), for comparison.

From the first two columns in the Figure 6, one can see that both FCN and the proposed method achieve excellent agreements with human observers on the UKBB dataset, indicating both of them can be used interchangeably with manual measurements. For the other two datasets, by contrast, the proposed method achieves much better agreement than FCN, as the LOA between the proposed method and manual results is narrower. For example, for *LVM* on the ACDC dataset, the LOA between the proposed method and the manual approach is from 5.07 to −39.93 (MD = −17.43) while the LOA between the FCN and the manual method is from 3.45 to −64.66 (MD = −30.61) (see Figures 6O,P, respectively).

Finally, we calculate the Spearmanr's rank correlation coefficients (*r*^2^) of the five clinical parameters derived from the automatic segmentation using the proposed method and the manual segmentation, which are reported in Table 9. From the results, it can be observed that the clinical measurements based on the LV segmentation and the myocardium segmentation derived by our automatic model are highly positively correlated with the manual analysis (≥0.91), although the RV correlation coefficients on the ACDC dataset are relatively lower.

**Table 9:** Spearman's rank correlation coefficients of clinical parameters derived from the automatic measurements and the manual measurements on the three sets.

**Comparison**	**Test set**	***LV*_*EDV*_**	***LV*_*ESV*_**	**LVM**	***RV*_*EDV*_**	***RV*_*ESV*_**
Automatic vs. Manual	UKBB (*n* = 600)	0.97	0.91	0.93	0.96	0.91
Automatic vs. Manual	ACDC (*n* = 100)	0.97	0.94	0.96	0.79	0.83
Automatic vs. Manual	BSCMR-AS (*n* = 599)	0.94	0.92	0.92	–	–

*All segmentations are produced by the U-Net trained with the UKBB training set*.

*Each coefficient reported in this table has a P-value below 0.0001*.

## 5. Discussion

In this paper, we developed a general training/testing pipeline based on data normalization and augmentation for improving the generalizability of neural network-based CMR image segmentation methods. We also highlighted the importance of the network structure and capacity (section 4.1) as well as the data normalization and augmentation strategies (section 4.2) for model generalizability. Extensive experiments on multiple test sets were conducted to validate the effectiveness of the proposed method. The proposed method achieves promising results on a large number of test images from various scanners and sites even though the training set is from one scanner, one site (sections 4.3, 4.4). Besides, the network is capable of segmenting healthy subjects as well as a group of pathological cases from multiple sources although it had only been trained with a *small* portion of pathological cases.

The limitation of the current method (the UKBB model) is that it still tends to underestimate the myocardium especially when the size of the myocardium becomes larger (see points in the right part of Figure 6R). Again, we conclude this limitation is mainly due to the lack of pathological cases in the training set.

Besides, we found that the difference (bias) between the automatic measurements and the manual measurements in the cross-domain test sets: ACDC and BSCMR-AS, are more significant than the difference in the intra-domain set: UKBB test set. The larger bias may be caused by not only those challenging pathological cases we have discussed above, but also inter-observer bias and the inconsistent labeling protocols used in the three datasets. The evident inter-observer variability when delineating myocardial boundaries on apical and basal slices in a single dataset has been reported in Suinesiaputra et al. (19). In this study, however, there are three datasets which were labeled by three *different* groups of observers. Each group followed an independent labeling protocol. As a result, significant variations of RV labels and MYO labels on the basal planes among the three datasets are found. This inter-dataset inconsistency of the RV labels on basal planes has been reported in Zheng et al. (33). The mismatch of RV labels can partially account for the negative MD values for the RV measurements in the ACDC dataset (see Figure 6V). The differences in the labeling protocols together with inter-observer variability in different datasets pose challenges to evaluate the model generalizability across domains accurately.

In the future, we will focus on improving the segmentation performance of the neural network by increasing the diversity of the training data in terms of pathology. A promising way of doing it, instead of collecting more labeled data, is to synthesize pathological cases by transforming existing healthy subjects with pathological deformations. A pioneering work (34) in this direction has successfully transported pathological deformations from certain pathological subjects (i.e., HCM, DCM) to healthy subjects, which can help to increase the number of pathological cases. Similarly, one can also adopt other types of learning-based data augmentation approaches [e.g., generative adversarial network based data augmentation (35), adversarial data augmentation (36)] to improve the model robustness on challenging cases, generating more realistic and challenging images (e.g., apical/basal slices, images with different types of artifacts) for the network to learn. Another direction, is to add a post-processing module to correct those failed predictions with anatomical constraints (37, 38). Both of these approaches can be easily integrated in the proposed training pipeline without significant modifications. Last but not least, for clinical deployment, it is necessary to alert users when failure happens. In this regard, future work can be integrating the segmentation approach with an automatic quality control module, providing automatic segmentation assessment [e.g., estimated segmentation scores (39), model uncertainty maps (40)] to clinicians for further verification and refinement.

## 6. Conclusion

In this paper, we proposed a general training/testing pipeline for neural network-based cardiac segmentation methods and revealed that a proper design of data normalization and augmentation, as well as network structure, play essential roles in improving its generalization ability across images from various domains. We have shown that a neural network (U-Net) trained with CMR images from a **single** scanner has the potential to produce competitive segmentation results on **multi-scanner** data across domains. Besides, experimental results have shown that the network is capable of segmenting healthy subjects as well as a group of pathological cases from multiple sources although it had only been trained with the UK Biobank data which has only a *small* portion of pathological cases. Although it might still have the limitations in segmenting images with low quality and some images with significant pathological deformations, higher segmentation accuracy for these subjects could be further achieved by increasing the diversity of training data regarding image quality and the pathology in the future.

## Data Availability Statement

The datasets generated for this study will not be made publicly available. However, the code for training and testing the segmentation network will be available at: https://github.com/cherise215/CardiacMRSegmentation. The code is used for data pre-processing, data augmentation, and the segmentation network training and testing. Researchers can apply to use the UK Biobank data resource for health-related research in the public interest on its website: www.ukbiobank.ac.uk/register-apply/. The ACDC data is open to the public and can be downloaded from its website: https://acdc.creatis.insa-lyon.fr/#challenges after registration. The BSCMR-AS dataset is available upon reasonable request.

## Ethics Statement

The UK Biobank data has approval from the North West Research Ethics Committee (REC reference: 11/NW/0382). The ACDC data is a publicly available dataset for cardiac MR image analysis which has approval from the local ethics committee of Hospital of Dijon (France). The BSCMR-AS data has approval from the UK National Research Ethics Service (REC reference:13/NW/0832), and has been conformed to the principles of the Declaration of Helsinki. All patients gave written informed consent.

## Author Contributions

CC, WB, and DR conceived and designed the study. RD, AB, JA, CM, and JM provided support on clinical aspects and they also provided the BSCMR-AS data resource to be used for testing. NA, AL, MS, KF, JP, SPe, EL, SPi, and SN provided the UKBB data resource to be used for training and testing and support on clinical aspects. EL and AB performed qualitative visual assessment of automated segmentation CC designed the method, performed data analysis, and wrote the manuscript. All authors read and approved the manuscript.

## Conflict of Interest

The authors declare that the research was conducted in the absence of any commercial or financial relationships that could be construed as a potential conflict of interest.

## Footnotes

^1^http://imaging.ukbiobank.ac.uk/

^2^https://www.creatis.insa-lyon.fr/Challenge/acdc/

^3^https://www.creatis.insa-lyon.fr/Challenge/acdc/evaluation.html

^4^https://acdc.creatis.insa-lyon.fr/description/databases.html

^5^https://pandas.pydata.org/

^6^https://docs.scipy.org/doc/scipy/reference/tutorial/stats.html

^7^https://www.statsmodels.org/stable/index.html

## Abbreviations

ACDC: automatic cardiac diagnosis challenge
ARV: abnormal right ventricle
AS: aortic stenosis
bSSFP: balanced steady-state free precession
BSCMR: the British society of cardiovascular magnetic resonance
CMR: cardiac magnetic resonance
CNN: convolutional neural network
DCM: dilated cardiomyopathy
ED: end-diastole
EDV: end-diastolic volume
ES: end-systole
ESV: end-systolic volume
FCN: fully convolutional network
GPU: graphics processing unit
HCM: hypertrophic cardiomyopathy
MD: mean difference
MICCAI: international conference on medical image computing and computer-assisted intervention
MINF: myocardial infarction with altered left ventricular ejection fraction
MR: magnetic resonance
MYO: myocardium
NOR: without cardiac disease
LOA: limits of agreement
LV: left ventricle
LVM: left ventricular mass
RV: right ventricle
SD: standard deviation of mean difference
SGD: stochastic gradient descent
SNR: signal-to-noise
UDDA: unsupervised deep domain adaptation
UKBB: UK Biobank.
