# Supplementary material for: Improving the Generalizability of Convolutional Neural Network-Based Segmentation on CMR Images
Source: Front Cardiovasc Med. 2020 Jun 30;7:105. doi: 10.3389/fcvm.2020.00105 (PMC7344224; doi:10.3389/fcvm.2020.00105)
Supplement: Supplementary file 1 [file Data_Sheet_1.PDF]

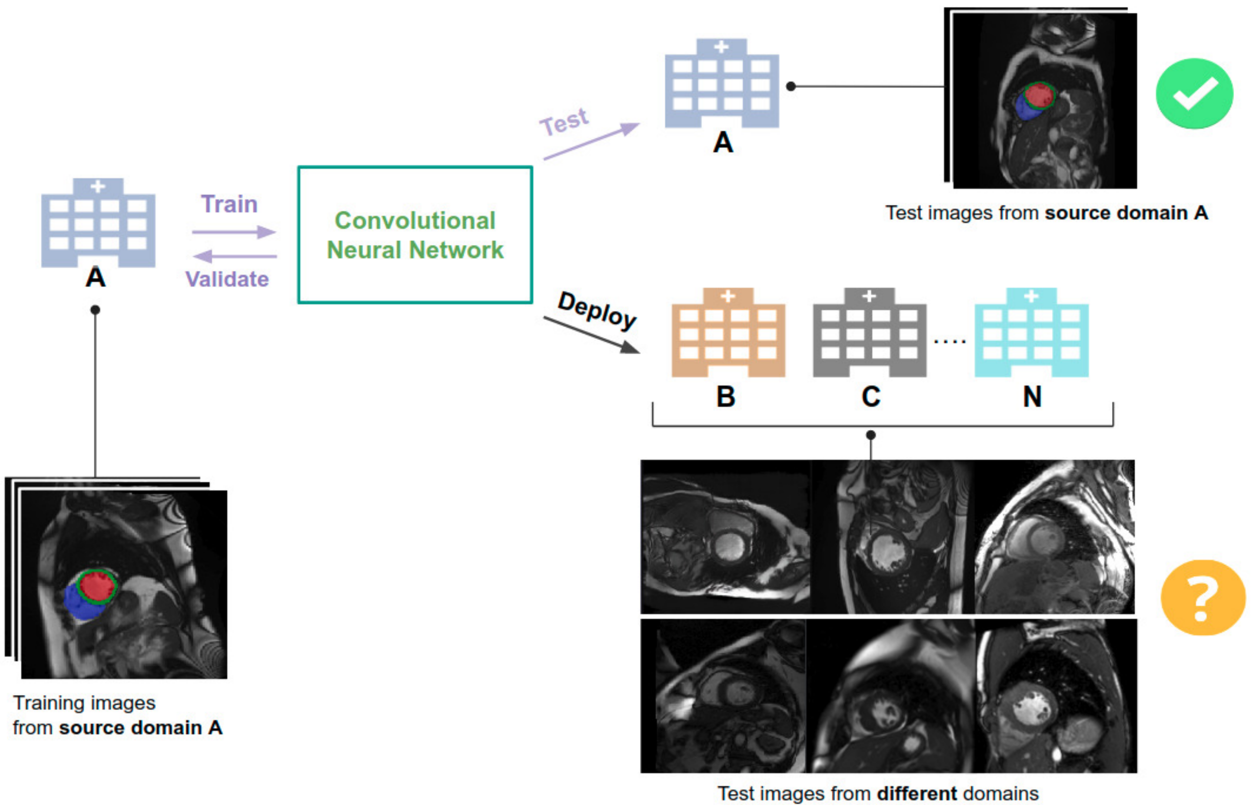

Figure S1. Illustration of a cross-domain CMR image segmentation application. A CNN model which has been trained using a dataset collected from site A (source domain) is deployed onto data from other sites (target domains) to segment the left ventricle, the myocardium and the right ventricle from CMR images. In general, the model can perform well on test images from the same domain. However, whether this model can generalise well onto other sites is unknown. For example, the training set may have limited pathological cases, which may cause the model not be able to generalise over subjects with heart conditions outside of the training set. In addition, images from different scanners may have different image appearance because of different imaging acquisition protocols. Both these differences pose challenges to applying a CNN-based cardiac segmentation model to everyday clinical practice.

Table S1. Segmentation performance across images of different slice thicknesses. The average Dice scores and standard deviation are reported for each group. The segmentation network was trained on images of 8 mm slice thickness.

Test dataset	Slice thickness (mm)	Number of images	LV		MYO		RV	
			mean	std	mean	std	mean	std
ACDC	5	24	0.89	0.06	0.76	0.09	0.82	0.09
	6.5	2	0.93	0.03	0.81	0.00	0.83	0.03
	7	2	0.85	0.09	0.69	0.05	0.82	0.06
	10	172	0.90	0.10	0.82	0.06	0.82	0.14
BSCMR-AS	5	4	0.89	0.08	0.85	0.04	-	-
	6	94	0.86	0.12	0.83	0.09	-	-
	7	486	0.88	0.1	0.83	0.07	-	-
	8	294	0.89	0.09	0.83	0.07	-	-
	10	318	0.89	0.07	0.85	0.04	-	-
